# Restoring cellular copper homeostasis in Alzheimer disease: a novel peptide shuttle is internalized by an ATP-dependent endocytosis pathway involving Rab5- and Rab14-endosomes

**DOI:** 10.3389/fmolb.2024.1355963

**Published:** 2024-04-05

**Authors:** Michael Okafor, Olivia Champomier, Laurent Raibaut, Sebahat Ozkan, Naima El Kholti, Stéphane Ory, Sylvette Chasserot-Golaz, Stéphane Gasman, Christelle Hureau, Peter Faller, Nicolas Vitale

**Affiliations:** ^1^ Institut des Neurosciences Cellulaires et Intégratives—Centre National de la Recherche Scientifique UPR3212, Université de Strasbourg, Strasbourg, France; ^2^ Institut de Chimie—UMR7177, Université de Strasbourg, Centre National de la Recherche Scientifique, Strasbourg, France; ^3^ Laboratoire de Chimie de Coordination, Centre National de la Recherche Scientifique UPR8241, Université de Toulouse, Toulouse, France; ^4^ Institut Universitaire de France (IUF), Paris, France

**Keywords:** Alzheimer’s disease, copper homeostasis, endocytosis, endolysosomes, rab GTPase

## Abstract

CPPs, or Cell-Penetrating Peptides, offer invaluable utility in disease treatment due to their ability to transport various therapeutic molecules across cellular membranes. Their unique characteristics, such as biocompatibility and low immunogenicity, make them ideal candidates for delivering drugs, genes, or imaging agents directly into cells. This targeted delivery enhances treatment efficacy while minimizing systemic side effects. CPPs exhibit versatility, crossing biological barriers and reaching intracellular targets that conventional drugs struggle to access. This capability holds promise in treating a wide array of diseases, including cancer, neurodegenerative disorders, and infectious diseases, offering a potent avenue for innovative and targeted therapies, yet their precise mechanism of cell entry is far from being fully understood. In order to correct Cu dysregulation found in various pathologies such as Alzheimer disease, we have recently conceived a peptide Cu(II) shuttle, based on the αR5W4 CPP, which, when bound to Cu(II), is able to readily enter a neurosecretory cell model, and release bioavailable Cu in cells. Furthermore, this shuttle has the capacity to protect cells in culture against oxidative stress-induced damage which occurs when Cu binds to the Aβ peptide. The aim of this study was therefore to characterize the cell entry route used by this shuttle and determine in which compartment Cu is released. Pharmacological treatments, siRNA silencing and colocalization experiments with GFP-Rab fusion proteins, indicate that the shuttle is internalized by an ATP-dependent endocytosis pathway involving both Rab5 and Rab14 endosomes route and suggest an early release of Cu from the shuttle.

## Introduction

Cell-penetrating peptides (CPPs) are short cationic or amphipathic peptides, typically consisting of 5–30 amino acids, with the remarkable ability to cross cell membranes. Discovered in the late 20th century, CPPs have since garnered immense attention in biomedical research for their potential applications in drug delivery, diagnostics, and therapeutics (Porosk and Langel, 2022). CPPs possess several distinctive properties that render them highly attractive for biomedical applications. Firstly, they exhibit a high degree of biocompatibility, meaning they are well-tolerated by biological systems, minimizing adverse reactions. This characteristic is crucial for their safe deployment in various therapeutic interventions. Additionally, CPPs demonstrate low immunogenicity, reducing the likelihood of provoking immune responses upon administration, thus ensuring their efficacy and safety in clinical settings. Another noteworthy feature of CPPs is their ability to efficiently traverse cell membranes, including those of eukaryotic cells, which are notoriously impermeable to most macromolecules. This unique capability allows CPPs to bypass the formidable barrier posed by the lipid bilayer, facilitating the delivery of cargo molecules, such as drugs, nucleic acids, or imaging agents, into the cytoplasm and even to specific organelles within the cell. Moreover, CPPs exhibit remarkable versatility in cargo delivery. They can transport a diverse array of therapeutic molecules, ranging from small chemical compounds to large biomacromolecules, across cellular membranes. This versatility opens numerous possibilities for targeted therapy, wherein CPPs can deliver therapeutic payloads precisely to diseased cells while sparing healthy ones, thereby enhancing treatment efficacy, and minimizing off-target effects.

The cellular entry mechanisms employed by CPPs have been the subject of extensive investigation, yielding valuable insights into their mode of action. While the precise mechanisms may vary depending on the CPP sequence, cargo molecule, and cell type, several common mechanisms have been proposed including direct translocation, endocytosis, receptor mediated uptake, and membrane fusion. One prevailing hypothesis suggests that certain CPPs possess inherent membrane-penetrating properties, allowing them to directly translocate across cell membranes without the need for specific receptors or energy-dependent processes (Madani et al., 2011). Depending on the physicochemical properties of CPPs, such as their charge, hydrophobicity, and secondary structure, several models have been proposed to explain the direct membrane transduction of CPPs, including the “carpet model,” where CPPs cover the cell membrane like a carpet, and the “barrel-stave model,” where CPPs form pores in the membrane. The interplay between CPPs and cell surface proteoglycans is also crucial in facilitating cellular uptake. Electrostatic interactions between the positively charged CPPs and negatively charged cell surface heparan sulfate proteoglycans contribute to the initial binding step, leading to enhanced internalization. Moreover, the cellular microenvironment, including pH, temperature, and the presence of serum proteins, can influence the efficacy of CPP-mediated delivery. Additionally, endocytosis, including clathrin- and caveolae-mediated pathways as well as macropinocytosis has been recognized as another significant pathway for CPP internalization (Madani et al., 2011). Endocytosis involves the formation of vesicles that engulf extracellular material to transport it into the cell. While progress has been made in elucidating the mechanisms of CPP cell entry, challenges and controversies persist. However, it was recently shown in HeLa cells knocked out for the KCNN4 channel to prevent CPP membrane translocation, that the five commonly used CPPs, TAT, R9, Penetratin, MAP and Transportan, use a non-classical endocytosis route (Trofimenko et al., 2021). This alternative entry route specifically involves a previously undescribed Rab5-and Rab7-independent pathway, but Rab14-positive endosomes ending in a non-acidic Lamp1 compartment (Trofimenko et al., 2021). Therefore, although CPPs represent a promising class of peptides with immense potential for revolutionizing pathology treatment through enhanced drug delivery and targeted therapy, continued research into their cellular entry mechanisms holds the key to unlocking their full therapeutic potential and advancing the field of biomedical science.

One of these therapeutic potential is its potential use in equilibrating Cu dyshomeostasis as seen in Alzheimer’s disease (AD) (Okafor et al., 2022). AD is the main source of dementia, responsible for 60%–70% of cases worldwide. In latest reports, 57.4 million people worldwide suffered from dementia in 2019, and 152.8 million are expected to do so by 2050 (Nichols et al., 2022). This is thought to be partially due to the drastic increase in life expectancy and the ageing of the baby-boom generation (Bondi et al., 2017). Hallmarks of the AD include deposits of aggregated proteins including Tau proteins, which form intracellular neurofibrillary tangles due to their hyperphosphorylation, and amyloid-beta peptides (Aβ), which form extracellular plaques. Accumulation of Aβ is the consequence of abnormal metabolism of a transmembrane protein, the amyloid precursor protein (APP), caused in part by an imbalance between two pathways: the non-amyloidogenic pathway and the amyloidogenic pathway (O’Brien and Wong, 2011). The balance between these two pathways, together with the degradation pathway are therefore essential to limit the amount of Aβ peptide produced.

In the Aβ accumulated in the brain, exists high concentrations of Cu ions, which have been shown to promote their stabilization and contributing to the production of ROS (Miller et al., 2006; Schrag et al., 2011; James et al., 2012; Rembach et al., 2013; Hureau, 2023). On the other hand, there is a reduced level of Cu when considering the entire brain of AD patients, with more reduction especially in regions affected by the disease. Thus among various other factors, it has been suggested that metal dyshomeostasis, in particular of copper (Cu), may play a role in the development of AD (An et al., 2022). Cu is an indispensable trace element in the human body, required for the functioning of numerous enzymes and the metabolism of neurotransmitters, several nutrients including sugar, lipids, and iron. The human body contains a very small amount of Cu (≈100 mg), stored mainly in the liver, heart, muscles, and brain (Falcone et al., 2021a). Nevertheless, this metal has many biological roles and is notably involved in ATP synthesis to supply cells with energy, in the nervous (neurotransmitter synthesis and secretion, myelin production), immune and hematopoietic systems, bone mineralization, as well as in cellular protection against free radicals (Falcone et al., 2021a).

The development of monoclonal antibodies to prevent Aβ-related damages in the brain, thereby reducing brain toxicity and slowing disease progression has been a major focus of the pharmaceutical industry over the last 2 decades. Although some of these antibodies have shown signs of plaque clearance, their effectiveness in preventing disease progression and cognitive decline remains lacking (van Dyck et al., 2023). One likely explanation for this failure may be because other aspects of the disease have been neglected, one of which might be to restore normal brain Cu homeostasis in AD (Mehta et al., 2017). To this end, we have conceived and characterized a peptide Cu(II) shuttle, AKH-αR5W4^NBD^, that is capable of capturing and importing Cu into neuronal cells to restore normal Cu metabolism (Okafor et al., 2022). This peptide consists of a Cu(II)-binding domain, a cell penetrating peptide (CPP) domain, completed by a fluorophore for visualizing the Cu(II)-shuttle (Supplementary Figure S1). Our recent work has shown that this Cu(II) shuttle readily enters a neurosympathetic cell model. It was also observed that this Cu(II) shuttle i) retrieves Cu from Aβ and prevents associated ROS production, ii) rescues cells in culture from oxidative stress induced by Aβ-Cu, and iii) imports the Cu extracted from Aβ into these cells (Okafor et al., 2022). In cells it was detected in multiple vesicular structures that remain to be characterized (Okafor et al., 2022). Investigating its entry and transit within cells could allow modifications of cellular Cu levels and thus contribute to a deeper understanding of the regulation of Cu homeostasis in the body and help in the development of therapies against Cu-related diseases.

## Results

**FIGURE 1:**
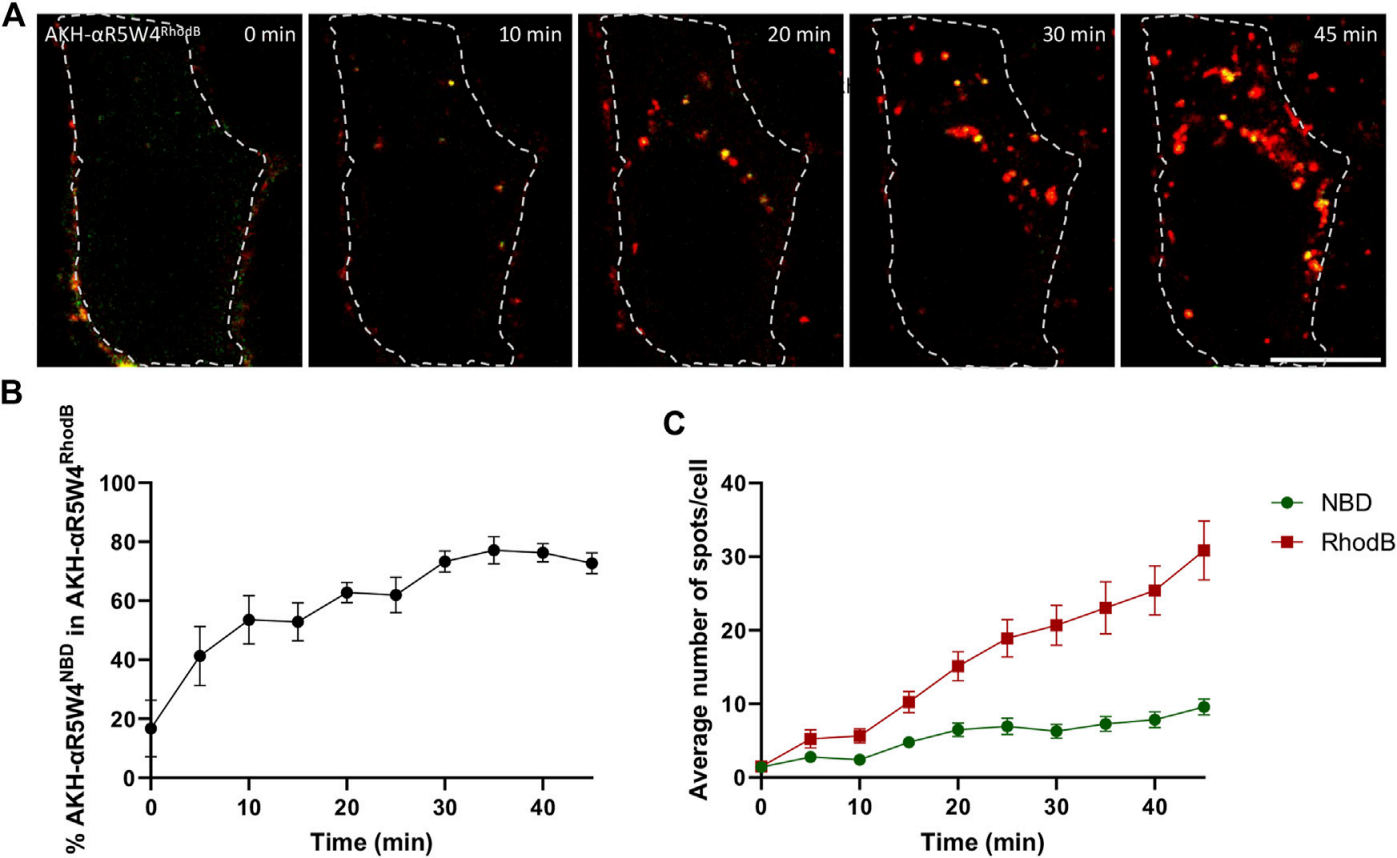
AKH-αR5W4^RhodB^ and AKH-αR5W4^NBD^ are found in the same vesicular compartments. **(A)** Representative images of a cell treated with 5 µM AKH-αR5W4^RhodB^ and 5 µM AKH-αR5W4^NBD^ for 5 min before a chase of 45 min at 37°C. Cell outlines used for quantifications are shown. Time zero represents 5 min after the pulse. Scale bar: 10 µm. **(B)** Percentage of AKH-αR5W4^NBD^ containing vesicles (spots) colocalized with spots containing AKH-αR5W4^RhodB^. **(C)** Average number of spots containing AKH-αR5W4^NBD^ or AKH-αR5W4^RhodB^. The data corresponds to the mean ± SEM, of three independent experiments with >30 cells analyzed.

HeLa cells (human cells derived from cervical cancer tumors) is a model of choice in cell biology for the study of intracellular vesicular trafficking (Lucey et al., 2009) and has been used previously to characterize the endocytic route of various CCP (Trofimenko et al., 2021). As colocalization studies with GFP-labeled markers were planned, we first compared the cell entry kinetics of the AKH-αR5W4 peptide labeled either with the green-fluorescent dye NBD or the red-fluorescent dye Rhodamine B (Figure 1). When both peptides were simultaneously added to HeLa cells, live image acquisition revealed vesicular structures positive for these shuttles close to the plasma membrane at early time points, with additional perinuclear vesicular structures in the center of the cell at later time points, in agreement with a classical endocytosis route. The number of vesicular structures positive for the Rhodamine B- and NBD-labelled peptide shuttle increased linearly with time and colocalized at different time points (Figures 1B, C). Interestingly, AKH-αR5W4^NBD^ positive structures were seen at earlier time points than AKH-αR5W4^RhodB^ positive ones and AKH-αR5W4^RhodB^ entry was greater than AKH-αR5W4^NBD^ after 5 min of chase. This may be the result of more membrane binding of AKH-αR5W4^RhodB^ during the pulse step thanks to the higher lipophilicity of Rhodamine B fluorophore compared to NBD (Magalhães et al., 2022).

It has been shown in the literature that peptides containing cell penetrating motifs are capable of crossing the cell membrane through direct translocation by local destabilization of the membrane lipid bilayer or by endocytosis (Liu et al., 2022). Although endocytosis can be driven by various proteins, including clathrin or caveolin proteins, all endocytic processes require ATP consumption to proceed. Intriguingly, a recent work suggests that cationic substances, including positively charged CPP, enter cells by an unusual ATP-dependent endocytic pathway that involves Rab14 positive endosomes (Trofimenko et al., 2021). To determine whether our Cu-shuttle crosses the plasma membrane or uses an energy-dependent mechanism to enter the cell, we carried out pharmacological studies aimed at reducing ATP availability and assessing its impact on Cu-shuttle endocytosis.

**FIGURE 2:**
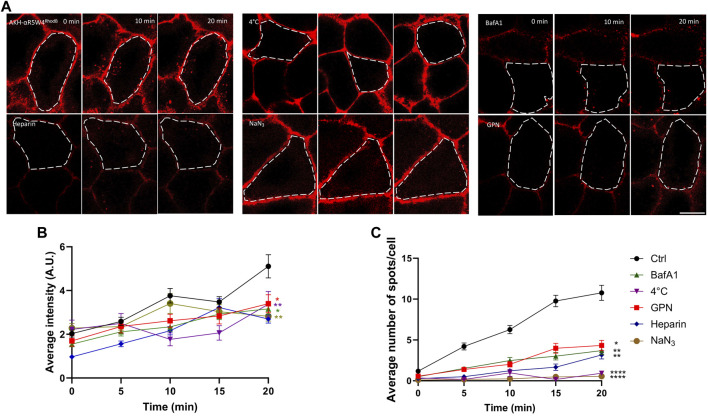
Penetration of AKH-αR5W4^RhodB^ in Hela cells is ATP dependent. **(A)** Cells were pretreated for 1 h in Locke solution containing 150 µM sodium azide (NaN_3_), 5 µM glycyl-l-phenylalanine 2-naphthylamide (GPN), 5 µM Bafilomycin A1 (BafA1), or with Locke solution at 4°C, before a pulse of 1 min with 10 µM AKH-αR5W4^RhodB^. For the Heparin condition, 0.5 U/mL heparin was mixed directly with AKH-αR5W4^RhodB^ before a 1-min pulse. Scale bar: 10 µm. Cell outlines used for quantifications are shown. **(B)** Quantification of the average cell fluorescence intensity, and **(C)** number of vesicles containing AKH-αR5W4^RhodB^ over 20 min. A non-parametric Kruskal–Wallis with Dunn’s multiple comparisons test was performed, *p < 0.01, **p < 0.001 and ****p < 0.00001. The data corresponds to the mean ± SEM, of three independent experiments with >30 cells analyzed.

Although all the treatments used here significantly decreased the intracellular fluorescence intensity after 20 min, a greater variability of the fluorescence intensity was seen at time zero and at subsequent time points (Figure 2B). Thus, to get a more robust analysis we focused on the number of vesicular structures detected. When temperature is lowered, the metabolic activity of cells decreases, resulting in a slowdown of endocytosis. In the context of our experiment, we observed very low vesicular labeling of AKH-αR5W4^RhodB^ when cells were incubated at 4°C, indicating a lack of peptide internalization for the duration of the recordings. This suggests that AKH-αR5W4^RhodB^ peptide entry into cells is likely to depend on cellular metabolic activity. To further validate this observation, sodium azide (NaN_3_), an inhibitor of cytochrome c oxidase forming part of the mitochondrial respiratory chain (Finch and Chan, 1996) was tested on Hela cells. By blocking this enzyme, NaN_3_ disrupts cellular respiration and reduces ATP production by lowering the proton-motive force (Bowler et al., 2006). Virtually no intracellular vesicular structures positive for AKH-αR5W4^RhodB^ were observed in cells after treatment with NaN_3_ (Figure 2A), suggesting that Cu-shuttle entry is largely ATP-dependent. These findings corroborate the observations made in low temperature conditions and highlight the essential role of ATP in the cellular entry of our Cu-shuttle, underlining the importance of cellular energy in regulating this entry pathway.

Next, we investigated the impact of impairing the lysosomal degradative pathway on AKH-αR5W4^RhodB^ cell penetration and traffic. Bafilomycin A1 (BafA1) is a chemical compound in the macrolide antibiotic family, produced from a variety of streptomycetes, which acts as a specific inhibitor of vacuolar H^+^ ATPase (V-ATPase), an enzyme responsible for the acidification of cellular compartments such as endosomes, lysosomes and secretory vesicles (Bowman et al., 1988). By blocking V-ATPase function, BafA1 disrupts intracellular acidification by inhibiting the flow of protons across membranes, leading to an increase in pH in intracellular compartments (Wang et al., 2021). This inhibition affects maturation of most endosomal compartments and has thus various consequences on cellular processes, including endocytosis, lysosomal degradation, autophagy, and intracellular trafficking of vesicles which are slowed down (Mauvezin et al., 2015). We observe that pre-treatment of HeLa cells with the endolysosomal vesicular ATPase inhibitor, prior to addition of the Cu-shuttle, has a significant impact on AKH-αR5W4^RhodB^ peptide entry (66% reduction at 20 min incubation) suggesting that this entry pathway is also dependent on acidification of the endolysosomal compartments (Figure 2). GPN (Glycyl-L-phenylalanine-β-naphthylamide) is another commonly used molecule that disrupts lysosomes. It is hypothesized that GPN is cleaved by the lysosomal enzyme cathepsin C, leading to disruption of lysosomal membranes, thus creating favorable experimental conditions for studying the effects of lysosome disruption and function. GPN causes a sustained increase in lysosomal pH and transient increases in cytosolic pH and Ca^2+^ concentration (Atakpa et al., 2019). Accordingly, GPN is widely used as a tool to explore endocytosis pathways, protein degradation and other processes involving lysosomes (Berg et al., 1994). An approximately three-fold reduction in the number of AKH-αR5W4^RhodB^-positive vesicles is observed (Figure 2). These observations suggest that normal lysosomal function might be required for the entry mechanism of our Cu-shuttle.

Given that entry of AKH-αR5W4^RhodB^ by endocytosis is ATP-dependent and needs the normal lysosome function, we wondered if the binding of the peptide was selective to a surface receptor, or if AKH-αR5W4^RhodB^ made membrane contact with negatively charged molecules on the membrane (ex: heparan sulfate) thanks to its polycationic charge. For this, competition with a homologue of heparan sulfate, heparin, was carried out. Heparin is a naturally occurring sulfated polysaccharide that is commonly used as an anticoagulant. However, heparin can also disrupt electrostatic interactions, as cationic peptides often use electrostatic interactions with the negative charges of cell membranes to facilitate their entry (Capila and Linhardt, 2002). Here we observed a significant drop in peptide entry in the presence of heparin, confirming the hypothesis that the Cu-shuttle, AKH-αR5W4^RhodB^, uses electrostatic interactions to bind to cell membranes before entering cells (Figure 2).

After validating that the entry of AKH-αR5W4^RhodB^ was ATP-dependent and involved electrostatic interactions as well, we investigated through which endocytic pathway AKH-αR5W4^RhodB^ is transported into cells. For this, we overexpressed GFP-tagged Rab proteins (Rab-GFP) in Hela cells. Colocalization analysis with AKH-αR5W4^RhodB^ were used to define the trafficking pathways followed by our Cu-shuttle. Rab proteins are key players in the regulation of intracellular trafficking. Although these proteins are structurally similar, their specific localization and function make them good markers of vesicular compartments. For example, the classical endocytosis route that mediates the entry of transferrin, dextran, and EGF involves the sequential contribution of clathrin-coated vesicles and certain types of endosomes.

It is important to note that ectopic expression of fluorescent endosomal markers, in live cells in particular, was shown not to affect endocytic processes (Trofimenko et al., 2021). These routes reach initially Rab5-positive endosomes and then move alternatively to Rab11-and Rab7-positive endosomes. In addition, Rab5 participates in homotypic fusion between early endosomes, facilitating the transfer of molecules between these intracellular compartments (Bucci et al., 1992; Rink et al., 2005). Alternatively, a non-classical endocytosis route has been proposed recently for cationic substances, including CPPs (Trofimenko et al., 2021). This potential alternative entry route involves Rab14-positive endosomes ending in a non-acidic Lamp1 compartment (Trofimenko et al., 2021). Rab14 is found associated to a subset of early endosomes and at the trans-Golgi network and is proposed to play a role in the transport of material between early endosomes and Golgi (Bhuin and Roy, 2014). Finally, Rab1 has been involved in various cellular processes important for proper cell function, such as autophagy. The Rab1A isoform is a key regulator of early endosome sorting and this protein is also thought to be involved in the transport and processing of the APP protein in Alzheimer’s disease (Dugan et al., 1995; Mukhopadhyay et al., 2014).

**FIGURE 3:**
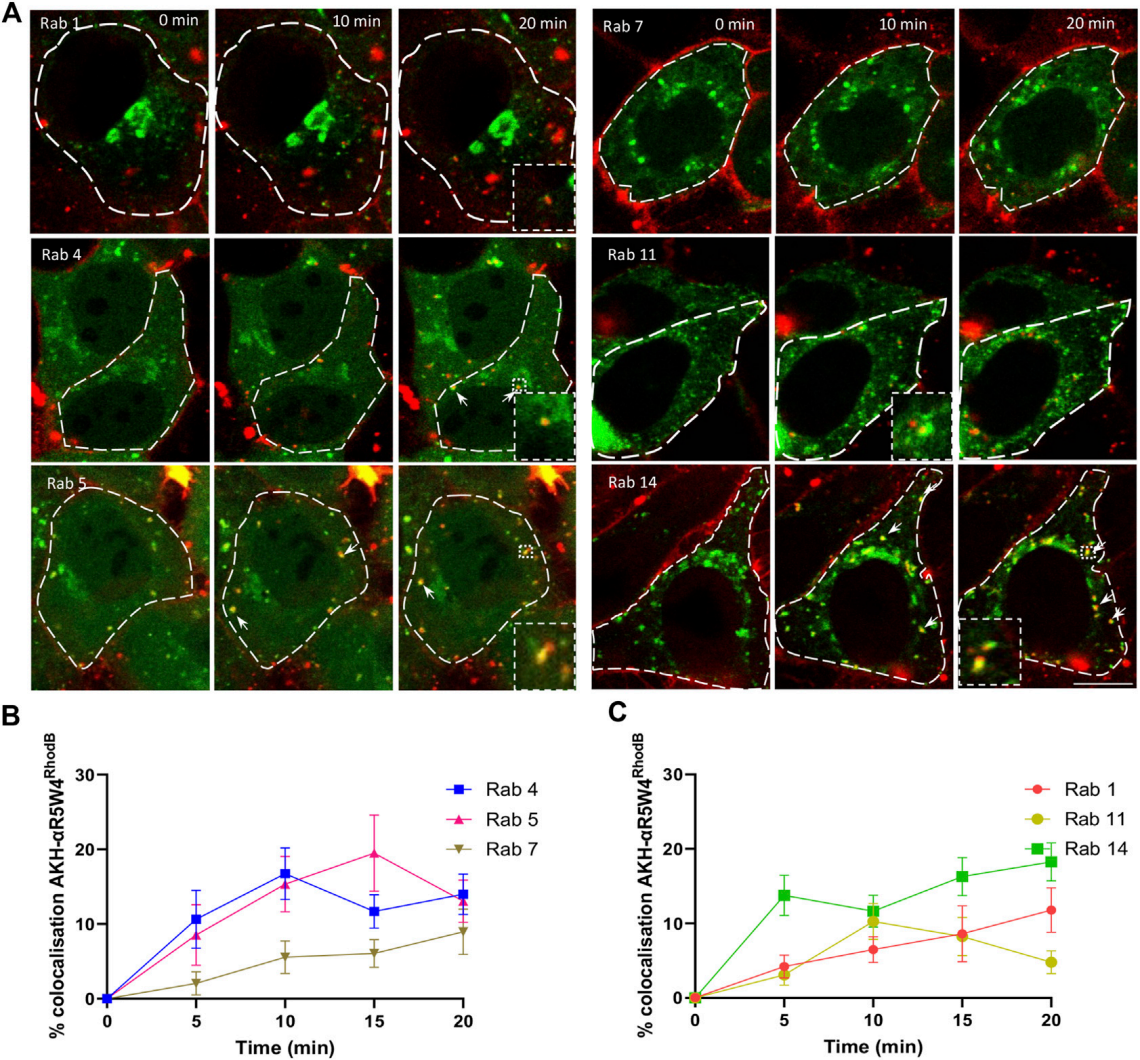
AKH-αR5W4^RhodB^ penetrates Hela cells through the classical endocytosis pathway and is detected in Golgi-derived vesicles. **(A)** Representative images of the colocalization between AKH-αR5W4^RhodB^ and exogenous Rab-GFP protein. Scale bar: 10 µm. Cell outlines used for quantifications are shown. **(B)** Quantification of the degree of colocalization between AKH-αR5W4^RhodB^ and Rab positive vesicles of the classical endocytic pathway or **(C)** with Golgi apparatus-related Rab proteins. The data corresponds to the mean ± SEM, of at least three independent experiments with >30 cells analyzed.

Therefore, we asked whether AKH-αR5W4^RhodB^ travelled through Rab5-, Rab14-and or Rab1-positive endosomes. After a short 1 min incubation with AKH-αR5W4^RhodB^ followed by a chase, a maximum of 19% colocalization of the peptide with Rab5 was detected at 15 min of chase, followed by a slight decrease, suggesting that a significant portion of the peptide reached Rab5-positive compartment during the first 15 min before being transferred to another endosomal compartment (Figures 3A, B). We also observed colocalization between our Cu-shuttle and Rab14-GFP, that reached 14% already at 5 min and remained close to 20% at 20 min of chase, underlining the fact that the Cu-shuttle uses as efficiently Rab14-and Rab5-mediated transport pathway to transit HeLa cells (Figures 3B, C). It supports the idea that Rab14 is involved in the peptide entry process and highlights the importance of this specific pathway in regulating CPP intracellular trafficking, but also that AKH-αR5W4^RhodB^ transits as well through Rab5-positive endosomes unlike it was established for other CPP (Trofimenko et al., 2021). Finally, the Cu-shuttle seems to be in the Rab1 compartments albeit at lower levels progressively reaching 10% colocalization after 20 min of chase (Figure 3C).

To determine whether AKH-αR5W4^RhodB^ could enter the vesicular recycling pathway, we monitored its colocalization with Rab4 and Rab11. Rab4 plays a role in the fast recycling of receptors from early endosomes to the cell surface (van der Sluijs et al., 1992), whereas Rab11 is involved in the trafficking of molecules through perinuclear recycling endosomes, contributing to the slow recycling of molecules to the plasma membrane (Ullrich et al., 1996). A progressive increase in colocalization during the first 15 min is observed between AKH-αR5W4^RhodB^ and Rab4-GFP (Figure 3C). Given that Rab4 and Rab5 meet on a common subset of vesicle to coordinate cargo endocytosis and recycling (Sönnichsen et al., 2000), it is not surprising that both Rab proteins reach the same level of colocalization with AKH-αR5W4^RhodB^. On the other hand, a clear colocalization between the AKH-αR5W4^RhodB^ shuttle and Rab11-GFP was also observed with a peak after 10 min of chase which dropped thereafter, suggesting that the Cu-shuttle is also recycled via the slow recycling pathway involving the Rab11 protein (Figures 3A, C). Unfortunately, despite extensive acid washes we could not get completely rid of the plasma membrane staining of AKH-αR5W4^RhodB^ preventing us from performing classical recycling studies to validate the cell exit of endocytosed shuttle.

Some of the Rab proteins play a role in the formation and maturation of late endosomes/lysosomes. Among them, Rab7 plays a role in transport from early endosomes to late endosomes and from late endosomes to lysosomes by interacting with its effector protein Rab-interacting lysosomal protein (Cantalupo et al., 2001). Early endosomes are transformed into late endosomes by the action of Rab7, promoting endosome maturation and the sorting of molecules to lysosomes for degradation. Colocalization was observed to gradually increase within the cells during the first 20 min, suggesting that the Cu-shuttle is probably not immediately degraded by the cell at early stages (Figures 3A, C). Hence colocalization experiments with different Rab markers clearly suggest that AKH-αR5W4^RhodB^ traffics in cells via different endocytic routes.

**FIGURE 4:**
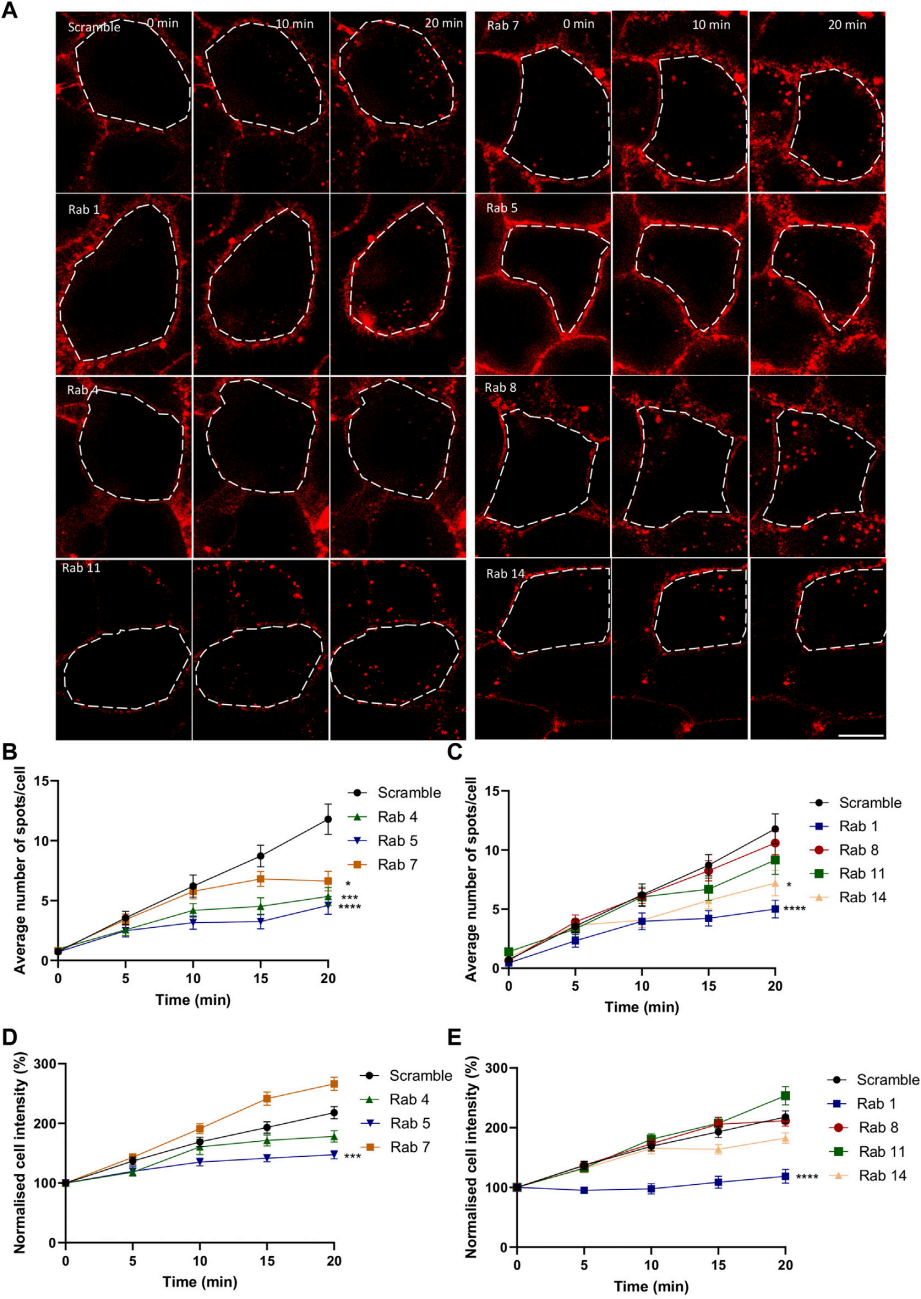
Silencing of Rab GTPases involved the classical endolysosomal pathway or transit through the Golgi apparatus attenuate AKH-αR5W4^RhodB^ cell entry. **(A)** Representative images showing the effect of selective Rab protein silencing by siRNA on AKH-αR5W4^RhodB^ internalization kinetics. Scale bar: 10 µm. Cell outlines used for quantifications are shown. Quantification of the number of vesicles **(B, C)**, and normalised average fluorescence intensity per cell containing AKH-αR5W4^RhodB^ over 20 min **(D, E)** after silencing of Rab GTPase involved in the classical endolysosomal pathway **(B, D)**, or Golgi related pathway **(C, E)**. A non-parametric Kruskal–Wallis with Dunn’s multiple comparisons test was performed, *p < 0.01, ***p < 0.0001 and ****p < 0.00001. The data corresponds to the mean ± SEM, of three independent experiments with >30 cells analyzed.

To further elucidate the role of each compartment, gene silencing with siRNA was employed. Since specific antibodies to validate siRNA efficiency on the numerous members of the Rab family members are lacking, we decided to use the ON-TARGETplus siRNA approach. These types of siRNA combine four different siRNA sequences and possess a modification pattern to achieve specificity for targeting a single gene, allowing a significant reduction in the concentration of individual siRNA used and therefore reducing off target effects. The efficiency of the siRNA was evaluated by testing the ability of Rab14 siRNA to prevent the overexpression of Rab14-GFP, which appeared to be greater than 90% (Supplementary Figure S2), suggesting that the siRNA used were highly efficient. Of note, for each Rab screened, an equimolar mixture of siRNA targeting their different isoforms was used concomitantly (Supplementary Table S1). Using a scrambled siRNA sequence as a control, we observed an almost linear increase over 20 min in the number of AKH-αR5W4^RhodB^-positive vesicles in HeLa cells (Figures 4B, C). In agreement with the colocalization observed above (Figure 3), inhibition of Rab5 protein expression reduced the number of vesicular structures detected at all time points tested and by 60% after 20 min chase (Figures 4A, B). Rab14 silencing also led to a progressive reduction in the number of AKH-αR5W4^RhodB^-labeled vesicular structures, but more moderately than with Rab5 silencing (Figures 4A, B). Rab14 silencing reduced by nearly 40% the number of vesicular structures positive for AKH-αR5W4^RhodB^ after 20 min chase (Figures 4A, C). In contrast, inhibiting the expression of the Rab11 protein resulted in a negligible decrease in the number of AKH-αR5W4^RhodB^-positive vesicles in HeLa cells. Notwithstanding, after 15min chase, there seems to be a modest decrease in vesicle number (Figure 4C), which is coherent given that it is involved in slow recycling (10–15 min). In agreement with the involvement of the lysosome in AKH-αR5W4^RhodB^ cell penetration, Rab7 silencing reduced the number of vesicular structures labeled by our AKH-αR5W4^RhodB^ after 15 min of incubation. More surprisingly, a sharp reduction in AKH-αR5W4^RhodB^ entry was also observed when Rab1 expression was reduced, which might thus result from functional changes throughout the endolysosomal system when Rab1 expression is altered. Finally, silencing of Rab8 involved in the Golgi to basolateral transport (Bhuin and Roy, 2014), had almost no effect on the entry and transport of the AKH-αR5W4^RhodB^, validating the specificity of these observations (Figure 4C). When considering the intracellular fluorescence intensity, both Rab5 and Rab1 silencing potently reduced AKH-αR5W4^RhodB^-positive signal in HeLa cells in agreement for an important role of both Rab5-and Rab1-positive vesicles in the entry route of our Cu-shuttle (Figures 4D, E). Silencing of Rab4, Rab8, Rab11, and Rab14 had a more moderate impact on the increase in AKH-αR5W4^RhodB^ signal over time (Figures 4D, E). Finally, Rab7 silencing led to an apparent increase compared to control condition of AKH-αR5W4^RhodB^ signal that did however not reach statistical significance (Figure 4D).

Complementary studies to characterize the entry route of the Cu-shuttle AKH-αR5W4 were performed in PC12 cells, the sympathetic neuronal cell model used in our previous study (Okafor et al., 2022). GPN, NaN_3_, and BafA1 treatments all led to a significant decrease in cell entry (Supplementary Figure S3), validating that in this neuronal cell model, the Cu-shuttle AKH-αR5W4 also mostly enters through an energy-dependent endocytosis pathway. Unfortunately, these cells have fewer vesicles positive for specific Rab endosomal markers per optical section than HeLa cells, which prevented us from performing a robust quantitative analysis and are thus illustrated here only by representative images. As observed in HeLa cells, some colocalization with the Rab4, 5, 7, 14 was seen as well (Supplementary Figure S4), suggesting the existence of multiple endocytosis routes also in this model.

**FIGURE 5:**
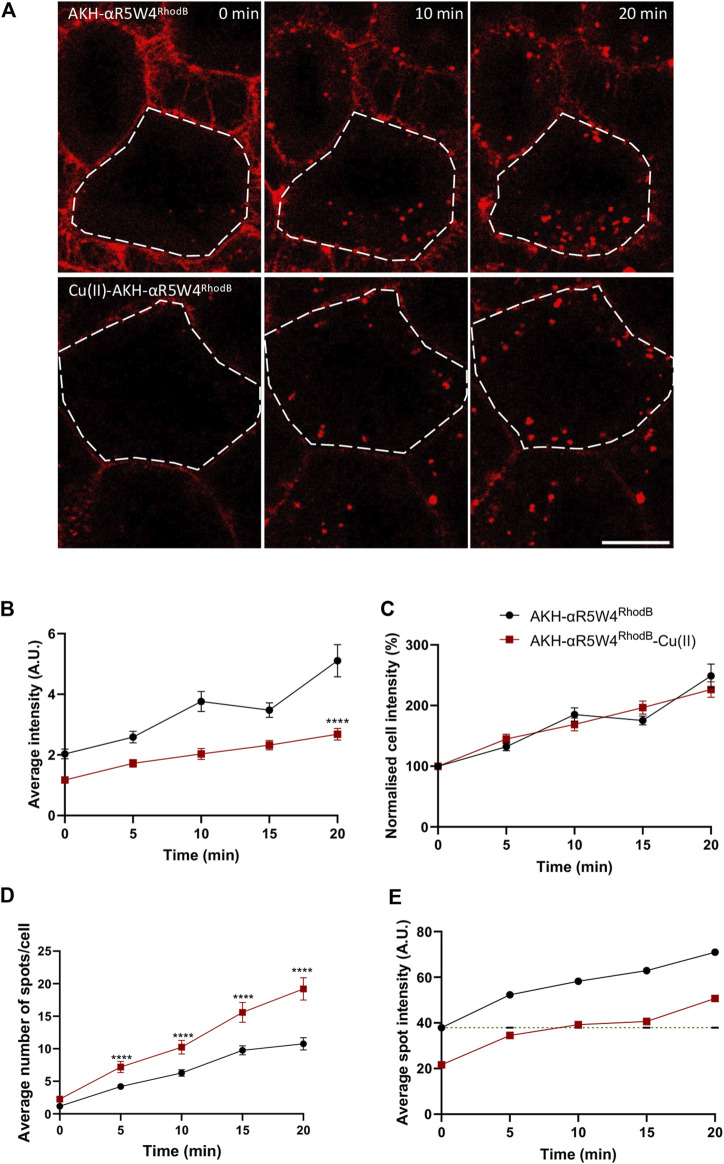
Complexation of AKH-αR5W4^RhodB^ with equimolar concentration of Cu, increases number of vesicles containing AKH-αR5W4^RhodB^. **(A)** Representative images of the effect of Cu on AKH-αR5W4^RhodB^ cell penetration. Scale bar: 10 µm. Cell outlines used for quantifications are shown. **(B)** Quantification of average cell fluorescence intensity, **(C)** normalised average cell fluorescence intensity. **(D)** Quantification of the number of vesicles per cell and **(E)** average fluorescence intensity of vesicles containing AKH-αR5W4^RhodB^ with or without Cu precomplexation. Header indicates average spot intensity in the absence of Cu at the zero time point. A non-parametric Mann-Whitney *t*-test was performed, ****p < 0.00001. The data corresponds to the mean ± SEM, of three independent experiments with >30 cells analyzed.

The proximity of the Cu(II) binding domain to the fluorophore induces partial quenching of the fluorescence when Cu(II) is bound to our shuttle. A quenching of approximatively 90% was quantified with AKH-αR5W4^NBD^ *in vitro* (Okafor et al., 2022). Given that Rhodamine B has a high extinction coefficient (Sil et al., 2018), despite the fact that its fluorescence is 90% quenched by Cu(II), the residual fluorescence is detectable. When comparing cells treated with the shuttle in the presence or in the absence of bound Cu, we observed a decrease by nearly 40% of the mean fluorescence intensity or number of vesicles containing AKH-αR5W4^RhodB^ when Cu is pre-complexed (AKH-αR5W4^RhodB^-Cu(II)) to the Cu-shuttle at all time points (Figures 5A, B, D). Of note normalization of the fluorescence intensity to 100% at time zero for both conditions led to an increase with very similar kinetics (Figure 5C). This justifies that Cu does not lead to decreased entry kinetics of the AKH-αR5W4^RhodB^ peptide. However, the pre-complexation of Cu to AKH-αR5W4^RhodB^, significantly increases the number of vesicles containing AKH-αR5W4^RhodB^ (Figures 5A, D). Interestingly, it takes 10 min for the average spot intensity of AKH-αR5W4^RhodB^-Cu to reach initial intensity of AKH-αR5W4^RhodB^. This observation could suggest that Cu dissociates from the shuttle progressively during this time frame, in the course of endosomes acidification.

## Discussion

This study was the first to investigate the transit mechanism of a novel shuttle, AKH-αR5W4, to restore Cu levels in a cellular model. We have previously shown that the AHKH^NBD^ short ATCUN peptide does not penetrate cells (Okafor et al., 2022), indicating that the ability of our Cu shuttle to penetrate cells relies primarily on its CPP motif. A recent study investigated the ability of various forms of Arg-Trp rich CPP peptide to penetrate CHO cells (Walrant et al., 2020). By playing with Arg/Trp ratio, it was found that the αR5W4 CPP peptide is by far the most efficient to enter cells through a CPP mode. They not only found that the residence time is longer, but also that for every endocytotic event, more αR5W4 molecules are associated to heparin sulfate thus explaining the deeper increase of internalization of this peptide compared to others. Altogether these data suggested that αR5W4 CPP peptide has the optimal sequence/structure/spatial arrangement (geometry) for efficient energetically favorable ionpair-π interactions involving Arg, Trp and negatively charged moieties on membranes (lipids, polysaccharides, or proteins). Our present results indicate that AKH-αR5W4^RhodB^ internalization in HeLa and PC12 cells follows an ATP-dependent endocytosis pathway, such as clathrin-mediated endocytosis, caveolin-mediated endocytosis or macropinocytosis. Indeed, endocytosis is an energetically costly and temperature-dependent phenomenon. When cells are exposed to a temperature of 4°C, metabolic activity is slowed, leading to a decrease in endocytosis of our Cu-shuttle (Figure 2). Furthermore, ATP depletion by NaN_3_ strongly reduced the internalization of the Cu-shuttle (Figure 2). Therefore, we can conclude that AKH-αR5W4 internalization is temperature- and ATP-dependent, and that it follows an ATP-dependent endocytosis pathway rather than penetrating directly across the plasma membrane. Altering the acidification of the endolysosomal compartment also led to a strong reduction of the Cu-shuttle cell entry (Figure 2) suggesting that proper function of this intracellular compartment is required for its efficient cell entry. This is however intriguing given that inhibiting the lysosomes usually leads to the accumulation of internalized molecules in the early endosomal compartments (Bayer et al., 1998; Haberger et al., 2020). However it has also been shown that inhibiting acidification could inhibit both receptor-mediated and fluid phase endocytosis (Xu et al., 2003). It is however of note that Rab7 silencing led to an apparent increase in fluorescence but not vesicle numbers (Figure 4), suggesting that hampering with the degradative pathway increases the size of AKH-αR5W4-positive vesicular structures. Classical pulse-chase experiments could have been envisioned to get more mechanistic information about the endocytosis process used by the AKH-αR5W4 peptide, for instance using BafA1. However, two technical drawbacks prevented us to perform such experiments. Firstly, BafA1 strongly impaired the peptide entry, which would impose adding BafA1 after the peptide. The second issue resides in the fact that BafA1’s action is rather slow and that a significant portion of the peptide remained bound to the plasma membrane (even despite acid washes) would continue to enter and likely progressively slowing down. Hence interpretation of these experiments would have been rather difficult to make as combining entry and trafficking parameters of the peptide.

**FIGURE 6:**
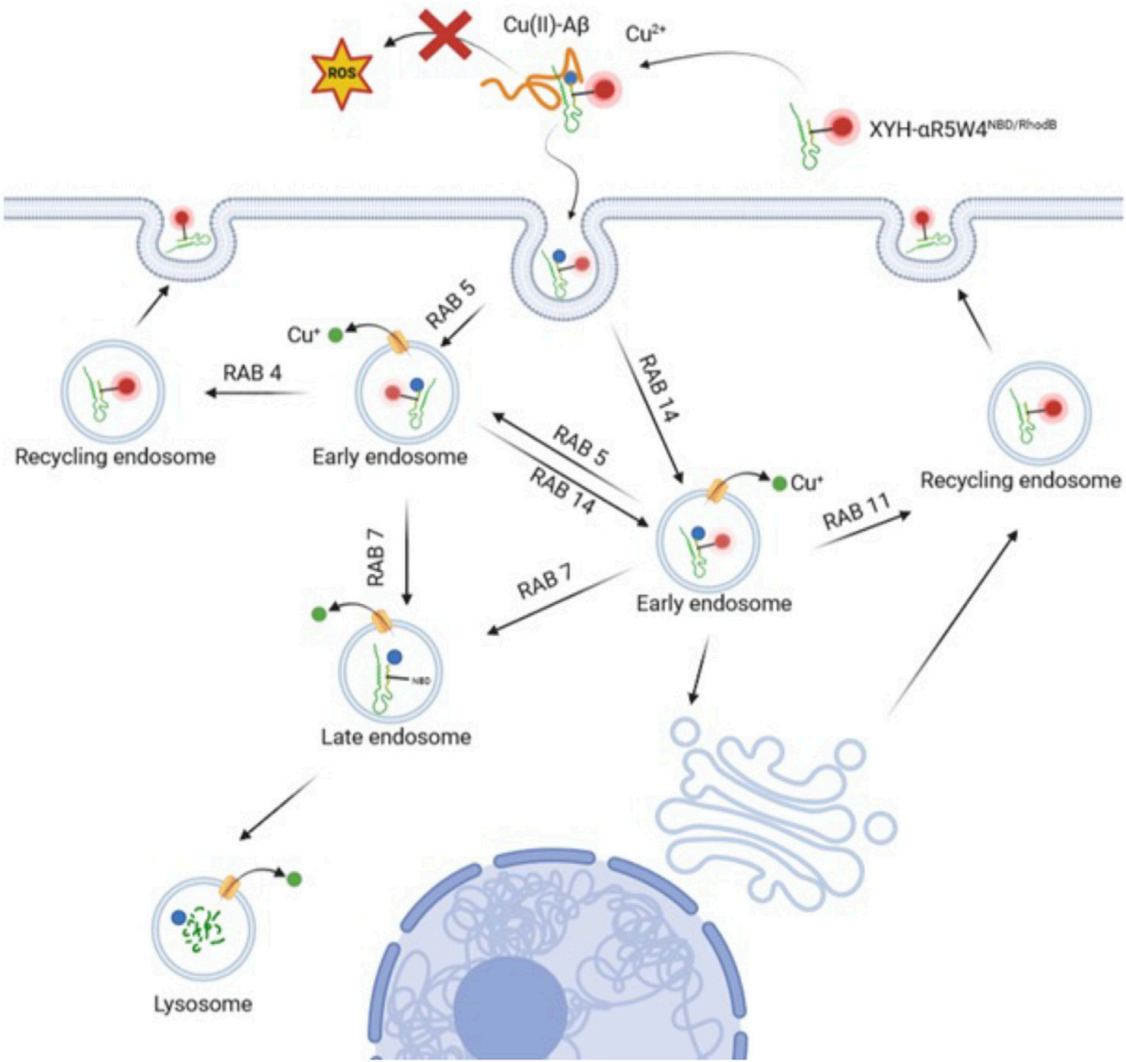
Model of the endocytic route for the Cu-shuttle AKH-αR5W4^RhodB^. The peptide AKH-αR5W4 captures Cu from Aβ aggregates thereby preventing ROS production and binds to the cellular membranes. The Cu-AKH-αR5W4^RhodB^ complex can then follow either Rab5 or Rab14 endocytic routes through which Cu dissociates from the shuttle. Specific endosomal transporters will vehicle Cu into the cytosol, whereas AKH-αR5W will be either recycled through Rab11 or Rab4 pathways or reach the degradative late endosomal/lysosomal compartment. Note that our silencing experiments suggest the existence of connections between the Rab5 and Rab14 pathways.

By using Rab GTPase overexpression and colocalization analysis, we determined through which compartments the Cu-shuttle travelled (Figure 6). Together our colocalization and silencing results suggest that, unlike what was reported recently for other positively charged peptides (Trofimenko et al., 2021), AKH-αR5W4 trafficking is not restricted to the unconventional Rab14 pathway but uses also the Rab5 pathway in HeLa cells. Presumably silencing together Rab5 and Rab14 could lead to a further reduction of the peptide entry compared to individually Rab silenced, confirming that the Cu-shuttle employs both Rab5 and Rab14 pathways. We did, however, not perform those experiments as it is also likely that a full blockage of the peptide’s entry into cells will not be seen, since it is expected that AKH-αR5W4 will probably be able to reach and accumulate in the early endosomal compartment. Since we observed little colocalization with Rab7 for the recorded time points, it is likely that entrance in the degradative pathway is modest and occurs only at later time points (Figure 3). Another fascinating observation is that in all silencing experiments, excepted for Rab7, we never observed an accumulation of AKH-αR5W4^RhodB^ in vesicular structures as would be anticipated if the entry route was a one-way road, suggesting that the peptide navigates through different alternative connected roads.

Surprisingly, albeit AKH-αR5W4 accumulates modestly in Rab1-positive endosomes over time, the silencing of Rab1 had a strong effect on internalization (plateaued after 10 mins of chase). The later may be explained by a defect in the motility of early endosomes caused by Rab1 silencing (Mukhopadhyay et al., 2014). Another explanation could be that the AKH-αR5W4 peptide navigates through the cellular endocytic compartments in an unspecific manner or that endocytosis is mediated through interaction between AKH-αR5W4 and positively charged molecules on the plasma membrane, such as Heparan sulfate proteoglycans as is the case for a large array of molecules (Holmes et al., 2013; Christianson and Belting, 2014). The latter seems plausible, as the competition with heparin greatly inhibits internalization of AKH-αR5W4 (Figure 2). The endocytic internalization of AKH-αR5W4 can be compared to that of the tetra-branched peptide known as NT4 or other polycationic CPPs, where the internalization mechanism seems promiscuous (Christianson and Belting, 2014; Mandarini et al., 2020). However, it should be noted that silencing of Rab8 or Rab11 did not significantly change the internalization of AKH-αR5W4, meaning that transport from Golgi apparatus to plasma membrane or the slow recycling of AKH-αR5W4 are not as important in the internalization mechanism of AKH-αR5W4. Nonetheless, our results show that the Cu-shuttle, AKH-αR5W4, is also transported by Rab11 and Rab4 positive vesicles with the latter potentially being the preferred recycling mechanism.

The presence of AKH-αR5W4 in Rab4 and Rab11 positive endosomes (Figures 3, 4), suggests that a significant portion may be recycled to the plasma membrane. Although this aspect needs to be further validated by different approaches, it may be of particular interest in terms of potential therapeutic use, as our Cu-shuttle may thus be capable of multiple cellular entries from the outer cellular space where it could capture Cu for delivery to the cytosol before degradation by the lysosome. It is also of importance to note that the mechanism of cell entry of AKH-αR5W4 dissected here in HeLa cells seems largely similar in the neuronal model PC12 cells. Further studies are however needed to test the ability of AKH-αR5W4 to enter the different neuronal cells, including neurons, astrocytes, and glial cells.

Finally, we show here that Cu probably dissociates from the shuttle within vesicular structures progressively, and not in a specific compartment (Figure 5). This dissociation is most likely to be induced by the progressive acidification that happens quickly at the early endosome stage. This is seen by the gradual increase of fluorescence intensity within the cells treated by Cu-AKH-αR5W4^RhodB^, signifying that the release of Cu is not compartment specific, but a steady process. This is supported by the fact that Cu(II) in the ATCUN motif starts to dissociate around pH = 6.5 (Maiti et al., 2020), similar to the pH of early endosomes (Murphy et al., 1984). We also excluded the possibility of a major Cu release due to reduction of Cu^2+^ to Cu^+^ in the cell by *in vitro* glutathione reduction (data not shown). In conclusion, these results support the robustness of AKH-αR5W4 internalization into cells, its processing through various endosomal compartments including the acidic Rab5 and non-acidic rab14 compartments and its use to study the mechanistic of Cu trafficking as well as a possible therapeutic agent.

## Materials and methods

### Material

All compounds used during this study were purchased from accredited merchants except stated otherwise. Dulbecco′s Modified Eagle’s Medium - high glucose (Sigma, D5796), horse serum (Gibco, 26050070), fetal calf serum (Gibco, 10270-160), penicillin/streptomycin (Sigma, P4458), trypsin (Gibco, 25300-054), siRNA (Dharmacon), DharmaFECT 1 (Dharmacon, T-2001-03).

### Peptide synthesis

Peptide synthesis was carried out according to general SPPS protocol (Falcone et al., 2021b). Peptide synthesis was carried out using the Biotage^®^ Initiator + Alstra™. A Fmoc-Rink-Amide Tenta XV RAM resin (charge of 0.24 mmol/g) was used for synthesis of all peptides. 0.5 M 1:1 ethyl cyano (hydroxyimino)acetate (Oxyma)/diisopropylcarbodiimide (DIC) was used for activation, 2 M DIEA dissolved in N-Methyl-2-pyrrolidone (NMP) was used as base. 5 M anhydride acetic acid was used to cap unreacted free amino groups.

The reaction time was 30 min coupling at ambient temperature, repeated once or 5 min coupling (repeated once) when using microwave (µWF) for compatible amino acids at 75°C except for cysteine, basic amino acids, and glycine after acidic amino acids. Fmoc deprotection was carried out using µWF at 75°C. Lysine was added as Nε-allyloxycarbonyl-protected (Fmoc-Lys (Alloc)-OH) to allow specific sidechain deprotection. The Alloc protecting group of the lysine side chain is deprotected using tetrakis (triphenylphosphine)palladium (0), a palladium-based mechanism (Thieriet et al., 1997). The Lissamine Rhodamine B fluorophore is added to the free lysine by nucleophilic substitution of 4 eq Lissamine Rhodamine B in the presence of DIEA. Finally, deprotection was carried out using TFA in the presence of scavengers (H_2_O and Triisopropylsilane). The peptide sequence, HPLC chromatogram, and LC-MS spectra are available in Supplementary Figure S1. Purity was estimated to be greater than 91.5%.

### Cell culture

HeLa cells were split twice a week up to a maximum of 15 splits. Cells were culture in DMEM high glucose with 10% horse serum inactivated, 5% Fetal bovine serum (Decomplemented) and 10% Penicillin (5000 U/mL)/Streptomycin (5 mg/mL) and incubated at 37°C with 5% CO_2_. PC12 cells were grown as described previously (Tryoen-Tóth et al., 2013).

### Transfection

#### Rab proteins

For transfection, jetPEI^®^ was used due to an efficient transfection in Hela cells. 2 × 10^4^ Hela cells were plated the day before transfection in a glass bottom Ibidi chamber coverslip. For each transfection, plasmids (DNA) are complexed with jetPEI^®^ at a ratio of 1:2 and the protocol is followed according to the manufacturer’s instructions. After incubation with jetPEI^®^ a total of 0.3 µg of final DNA is added to each well and left for 24 h before live imaging.

#### siRNA

DharmaFECT 1 Transfection Reagent was used to transfect siRNA. For each Rab, equimolar concentration of each isoform-specific siRNA was mixed to give a final concentration of 5 µM of siRNA for each isoform. 2 µL of siRNA was added to a tube containing 40 µL serum free media. 2 µL of DharmaFECT 1 was added to a tube containing 40 µL serum free media. The two tubes were mixed for 20 min and added to 2 × 10^4^ Hela cells plated in a glass bottom Ibidi chamber coverslip with antibiotic free DMEM 10% horse serum inactivated, 5% fetal bovine serum. The cells were left for 48 h before live imaging.

### Live imaging

Experiments were carried out in a heated chamber and maintained at 37°C. Cells underwent a pulse for 1 min with 10 µM of AKH-αR5W4^RhodB^, after which the peptide was removed followed by washing with Locke solution (140 mM NaCl, 4.7 mM KCl, 2.5 mM CaCl_2_, 1.2 mM KH_2_PO_4_, 1.2 mM MgSO_4_, 11 mM glucose, 0.56 mM ascorbic acid, 0.01 mM EDTA and 15 mM HEPES, pH 7.4). Subsequently, cells were recorded (chased) in fresh Locke solution for 20 min. Images were acquired using Leica TCS SP5 II confocal microscope at an interval of 1 min.

#### Rab colocalization

Hela cells were pulsed for 1 min with 10 µM of AKH-αR5W4^RhodB^, after which the peptide was removed followed by washing with Locke solution and pulsed for 20 min. Images of 4096 × 4096 pixel size were acquired every minute at to give a final pixel size of 60 nm. The following video showing the internalization are available as Supplementary Material: HeLa Rab4 Vs. AKH-αR5W4^RhodB^, HeLa Rab5 Vs. AKH-αR5W4^RhodB^, HeLa Rab11 Vs. AKH-αR5W4^RhodB^, HeLa Rab14 Vs. AKH-αR5W4^RhodB^, PC12 Rab4 Vs. AKH-αR5W4^RhodB^, PC12 Rab5 Vs. AKH-αR5W4^RhodB^, PC12 Rab7 Vs. AKH-αR5W4^RhodB^, PC12 Rab14 Vs. AKH-αR5W4^RhodB^, PC12 AKH-αR5W4^RhodB^ + 1 µM BafA1, PC12 AKH-αR5W4^RhodB^ + 5 µM GPN, PC12 AKH-αR5W4^RhodB^ +1% NaN_3_, and PC12 AKH-αR5W4^RhodB^.

#### Pharmacology experiments

Cells are pretreated with pharmacological drugs in Locke solution and maintained at 37°C for 1 h. Prior to recording, the cells were pulsed with a solution containing the pharmacological drug as well as 10 µM of AKH-αR5W4^RhodB^ for 1 min followed by a chase of 20 min. Images of 1024 × 1024 pixel size were acquired every minute at zoom 2 to give a final pixel size of 120 nm.

### Spot detection and colocalization

Using Icy software, each cell’s cytoplasm was contoured to form an ROI, to avoid signal from AKH-αR5W4^RhodB^ at the cell membrane. The number of spots in the ROI were counted using spot detector plugin, and colocalization between green and red signals was carried out with two overlapping spots deemed colocalized at less than 3 pixels. Of note, a minimum of two spots per cell, positive for AKH-αR5W4^RhodB^, was required for the colocalization analysis. The colocalization data is expressed as number of vesicles positive for AKH-αR5W4^NBD^ in AKH-αR5W4^RhodB^ or for AKH-αR5W4^RhodB^ in Rab positive vesicles.

For measurements of the cell intensity, the average intensity of the pixels within the selected ROIs were measured, and then the values were normalized to time t0 to get normalized cell intensities.

### Statistical analysis

The number of experiments and repeats are indicated in figure legends. Normality of the data distribution was verified, and statistical analysis was performed with t-tests or with ANOVA test relative to the indicated control with the help of Graphpad Prism 10.

## Data Availability

The raw data supporting the conclusion of this article will be made available by the authors, without undue reservation.

## Supplementary material

SUPPLEMENTARY VIDEO S1 Colocalization between AKH-αR5W4^RhodB^ (Red) and Rab4-GFP protein (Green). Confocal microscopy of living HeLa cells transiently expressing Rab-GFP protein, pre-treated with 10 µM AKH-αR5W4^RhodB^ for 1 min and acquired every minute at 4096 × 4096 pixel. Time in minutes after washing out AKH-αR5W4^RhodB^ is indicated on the top left part of the video.

SUPPLEMENTARY VIDEO S2 Colocalization between AKH-αR5W4^RhodB^ (Red) and Rab5-GFP protein (Green). Confocal microscopy of living HeLa cells transiently expressing Rab-GFP protein, pre-treated with 10 µM AKH-αR5W4^RhodB^ for 1 min and acquired every minute at 4096 × 4096 pixel. Time in minutes after washing out AKH-αR5W4^RhodB^ is indicated on the top left part of the video.

SUPPLEMENTARY VIDEO S3 Colocalization between AKH-αR5W4^RhodB^ (Red) and Rab11-GFP protein (Green). Confocal microscopy of living HeLa cells transiently expressing Rab-GFP protein, pre-treated with 10 µM AKH-αR5W4^RhodB^ for 1 min and acquired every minute at 4096 × 4096 pixel. Time in minutes after washing out AKH-αR5W4^RhodB^ is indicated on the top left part of the video.

SUPPLEMENTARY VIDEO S4 Colocalization between AKH-αR5W4^RhodB^ (Red) and Rab14-GFP protein (Green). Confocal microscopy of living HeLa cells transiently expressing Rab-GFP protein, pre-treated with 10 µM AKH-αR5W4^RhodB^ for 1 min and acquired every minute at 4096 × 4096 pixel. Time in minutes after washing out AKH-αR5W4^RhodB^ is indicated on the top left part of the video.

SUPPLEMENTARY VIDEO S5 Pharmacological treatment on PC12 cells. Confocal microscopy of living PC12 cells, pre-treated for 45 mins with 1 µM BafA1, then 10 µM AKH-αR5W4^RhodB^ for 5 min and acquired every 30 s at 1024 × 1024 pixel. Time in minutes after addition of AKH-αR5W4^RhodB^ is indicated on the top left part of the video.

SUPPLEMENTARY VIDEO S6 Pharmacological treatment on PC12 cells. Confocal microscopy of living PC12 cells, pre-treated for 45 mins with 5 µM GPN, then 10 µM AKH-αR5W4^RhodB^ for 5 min and acquired every 30 s at 1024 × 1024 pixel. Time in minutes after addition of AKH-αR5W4^RhodB^ is indicated on the top left part of the video.

SUPPLEMENTARY VIDEO S7 Pharmacological treatment on PC12 cells. Confocal microscopy of living PC12 cells, pre-treated for 45 mins with 1% NaN_3_, then 10 µM AKH-αR5W4^RhodB^ for 5 min and acquired every 30 s at 1024 × 1024 pixel. Time in minutes after addition of AKH-αR5W4^RhodB^ is indicated on the top left part of the video.

SUPPLEMENTARY VIDEO S8 Confocal microscopy of living PC12 cells treated with 10 µM AKH-αR5W4^RhodB^ for 5 min and then acquired every 30 s at 1024 × 1024 pixel. Time in minutes after addition of AKH-αR5W4^RhodB^ is indicated on the top left part of the video.

SUPPLEMENTARY VIDEO S9 Colocalization between AKH-αR5W4^RhodB^ (Red) and Rab4-GFP protein (Green). Confocal microscopy of living PC12 cells transiently expressing Rab-GFP protein, pre-treated with 10 µM AKH-αR5W4^RhodB^ for 5 min and acquired every 30 s at 1024 × 1024 pixel. Time in minutes after washing out AKH-αR5W4^RhodB^ is indicated on the top left part of the video.

SUPPLEMENTARY VIDEO S10 Colocalization between AKH-αR5W4^RhodB^ (Red) and Rab5-GFP protein (Green). Confocal microscopy of living PC12 cells transiently expressing Rab-GFP protein, pre-treated with 10 µM AKH-αR5W4^RhodB^ for 5 min and acquired every 30 s at 1024 × 1024 pixel. Time in minutes after washing out AKH-αR5W4^RhodB^ is indicated on the top left part of the video.

SUPPLEMENTARY VIDEO S11 Colocalization between AKH-αR5W4^RhodB^ (Red) and Rab7-GFP protein (Green). Confocal microscopy of living PC12 cells transiently expressing Rab-GFP protein, pre-treated with 10 µM AKH-αR5W4^RhodB^ for 5 min and acquired every 30 s at 1024 × 1024 pixel. Time in minutes after washing out AKH-αR5W4^RhodB^ is indicated on the top left part of the video.

SUPPLEMENTARY VIDEO S12 Colocalization between AKH-αR5W4^RhodB^ (Red) and Rab14-GFP protein (Green). Confocal microscopy of living PC12 cells transiently expressing Rab-GFP protein, pre-treated with 10 µM AKH-αR5W4^RhodB^ for 5 min and acquired every 30 s at 1024 × 1024 pixel. Time in minutes after washing out AKH-αR5W4^RhodB^ is indicated on the top left part of the video.

## Abbreviations

Alzheimer’s disease, (AD); Amyloid-beta, (Aβ); Bafilomycin A1, (BafA1); Copper, (Cu); Glycyl-L-phenylalanine-β-naphthylamide, (GPN); Reactive oxygen species, (ROS); Sodium azide, (NaN_3_).
