# Supplementary material for: Restoring cellular copper homeostasis in Alzheimer disease: a novel peptide shuttle is internalized by an ATP-dependent endocytosis pathway involving Rab5- and Rab14-endosomes
Source: Front Mol Biosci. 2024 Apr 5;11:1355963. doi: 10.3389/fmolb.2024.1355963 (PMC11026709; doi:10.3389/fmolb.2024.1355963)
Supplement: Supplementary file 4 [file Table1.pdf]

Rab	Isoform	Target sequence
Rab 1	A	CAGCAUGAAUCCCGAAUAU
		GUAGAACAGUCUUUCAUGA
		GGAAACCAGUGCUAAGAAU
		UGAGAAGUCCAAUGUUAAA
	B	UGCAGGAGAUUGACCGCUA
		CCAGCGAGAACGUCAAUAA
		CGGUGGGAUCUGAGUAUAU
		GAAUAUGACUACCUGUUUA
Rab 4	A	GCUCAGGAGUGUGGUUGUU
		UACAAUGCGCUUACUAAUU
		GAUAAUAAAUGUUGGUGGU
		GAACGAUUCAGGUCCGUGA
	B	GCACUAUCCUCAACAAGAU
		AGAAUAAGUUCAAACAGGA
		AAUCAUGUCUCCUUCAUCA
		UCAGUGACGCGGAGUUAUU
Rab 5	A	GCAAGCAAGUCCUAACAUU
		UGACACUACAGUAAAGUUU
		GGAAGAGGAGUAGACCUUA
		AGAGUCCGCUGUUGGCAAA
	B	GGAGCGAUAUCACAGCUUA
		GAAAGUCAAGCCUGGUAUU
		CAACAAACGUAUGGUGGAG
		AAGCUGCAAUCGUGGUUUA
	C	UCAUUGCACUCGCGGUAA
		GAACAAGAUCUGUCAAUUU
		GCAAUGAACGUGAACGAAA
		GCUAAGAAGCUUCCCAAGA
Rab 7	A	CUAGAUAGCUGGAGAGAUG
		AAACGGAGGUGGAGCUGUA
		GAUGGUGGAUGACAGGCUA
		GGGAAGACAUCACUCAUGA
	B	GUAGGGCUCUGUCGAGGUA
		GAAACUCAUUAUCGUCGGA
		UCAAUGUGGUGCAAGCGUU
		GGAAGUAGCUCAAGGCUGG

Rab	Isoform	Target sequence
Rab 8	A	CAGGAACGGUUUCGGACGA
		GAAUUAAACUGCAGAUAUG
		GAACAAGUGUGAUGUGAAU
		GAACUGGAUUCGCAACAUU
	B	GCAAUUGACUAUGGGAUUA
		GAACAAUCACGACAGCGUA
		GAUCAAAGAAGACCAGUUU
		CGAUAGAACUAGAUGGAAA
Rab 11	A	GCAACAAUGUGGUUCCUAU
		CAAGAGCGAUAUCGAGCUA
		GUGCAGUGCUGUCAGAACA
		GAGAUUUACCGCAUUGUUU
	B	UAACGUAGAGGAAGCAUUC
		GAGUACGACUACCUAUUCA
		UCGCCAAGCACCUGACCUA
		CAACUUGUCCUUCAUCGAG
Rab14		GCUCUUAUGGUCUAUGAUA
		CAACUGCACCAUACAACUA
		GAAAAUGGCUUAUUGUUCC
		GUACAAGAAUAAUCGAAGU
Rab14 rat		GUAUAUUAUUAUCGGGGAU
		UCUGAAUGCUGCCGAGUCU
		CAACUACACCGUACAACUA
		GGUGUUGAAUUUGGUACAA
Scramble		UGGUUUACAUGUCGACUAA
		UGGUUUACAUGUUGUGUGA
		UGGUUUACAUGUUUUCUGA
		UGGUUUACAUGUUUUCCUA

Table S1: List of ON-TARGETplus siRNA against Rab proteins. All Rab siRNA targets Human DNA except noted otherwise.
