# Supplementary material for: Restoring cellular copper homeostasis in Alzheimer disease: a novel peptide shuttle is internalized by an ATP-dependent endocytosis pathway involving Rab5- and Rab14-endosomes
Source: Front Mol Biosci. 2024 Apr 5;11:1355963. doi: 10.3389/fmolb.2024.1355963 (PMC11026709; doi:10.3389/fmolb.2024.1355963)
Supplement: Supplementary file 13 [file Image1.pdf]

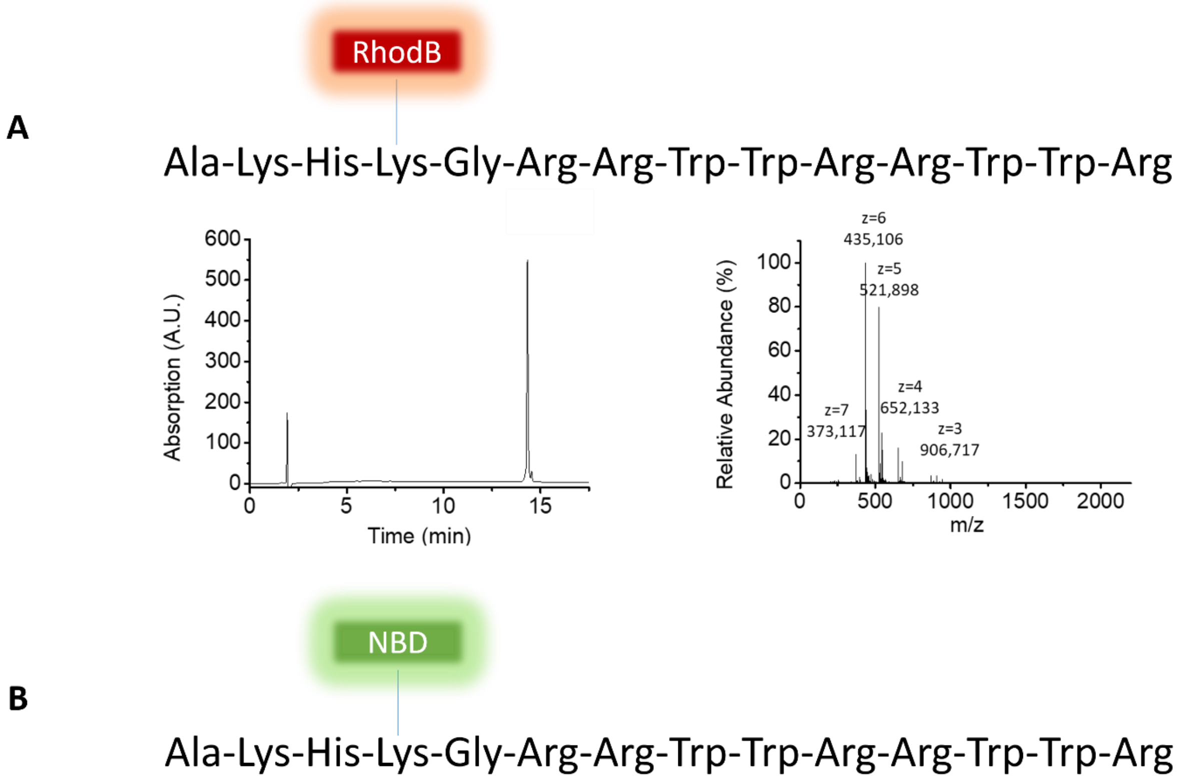

Figure S1: (A) Amino acid sequence along with HPLC chromatogram and LC-MS spectra of the Cu-shuttle AKH-αR5W4^{RhodB} and (B) AKH-αR5W4^{NBD} used in this study. .For HPLC, the separation was performed using a linear gradient of buffer A (TFA 0.1%) and buffer B (TFA 0.1%, ACN 90%) ranging from 5% buffer B to 100% buffer B in 30 mins, flow 1 ml/min, and UV detection at 214 nm. LC-MS spectra of purified. For LC-MS, the separation was performed using a linear gradient of buffer A (Formic acid 0.1%) and buffer B (Formic acid 0.1%, ACN 90%) ranging from 5% buffer B to 100% buffer B in 15 mins, flow 1 ml/min, and m/z detection from 0 to 2000 Da.

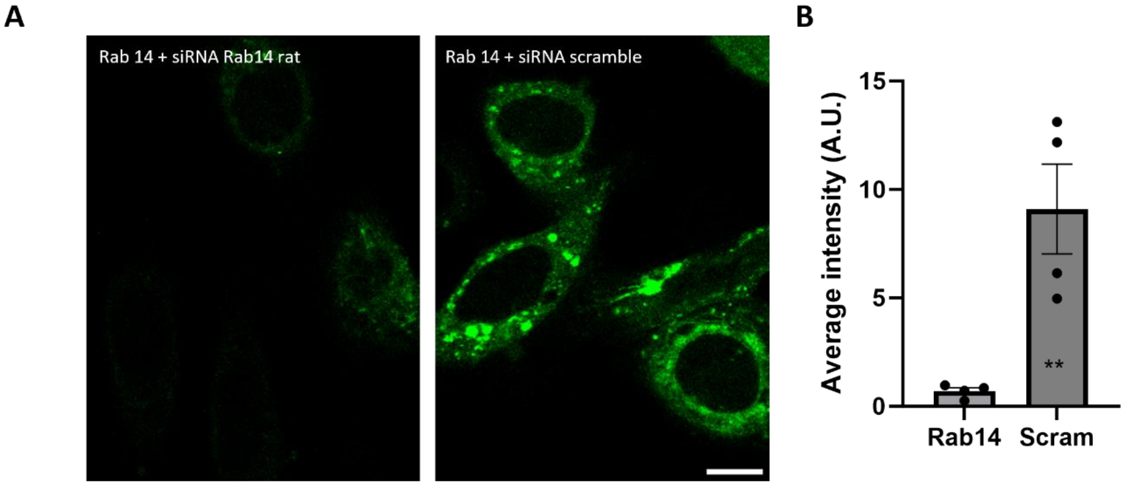

Figure S2: (A) Representative image of the effect of selective rat Rab14 siRNA vs scramble against transfected mouse Rab14-GFP protein translation. (B) Quantification of the average intensity of Rab14-GFP. 4 planes were averaged for each condition. Scale bar: 10 μm

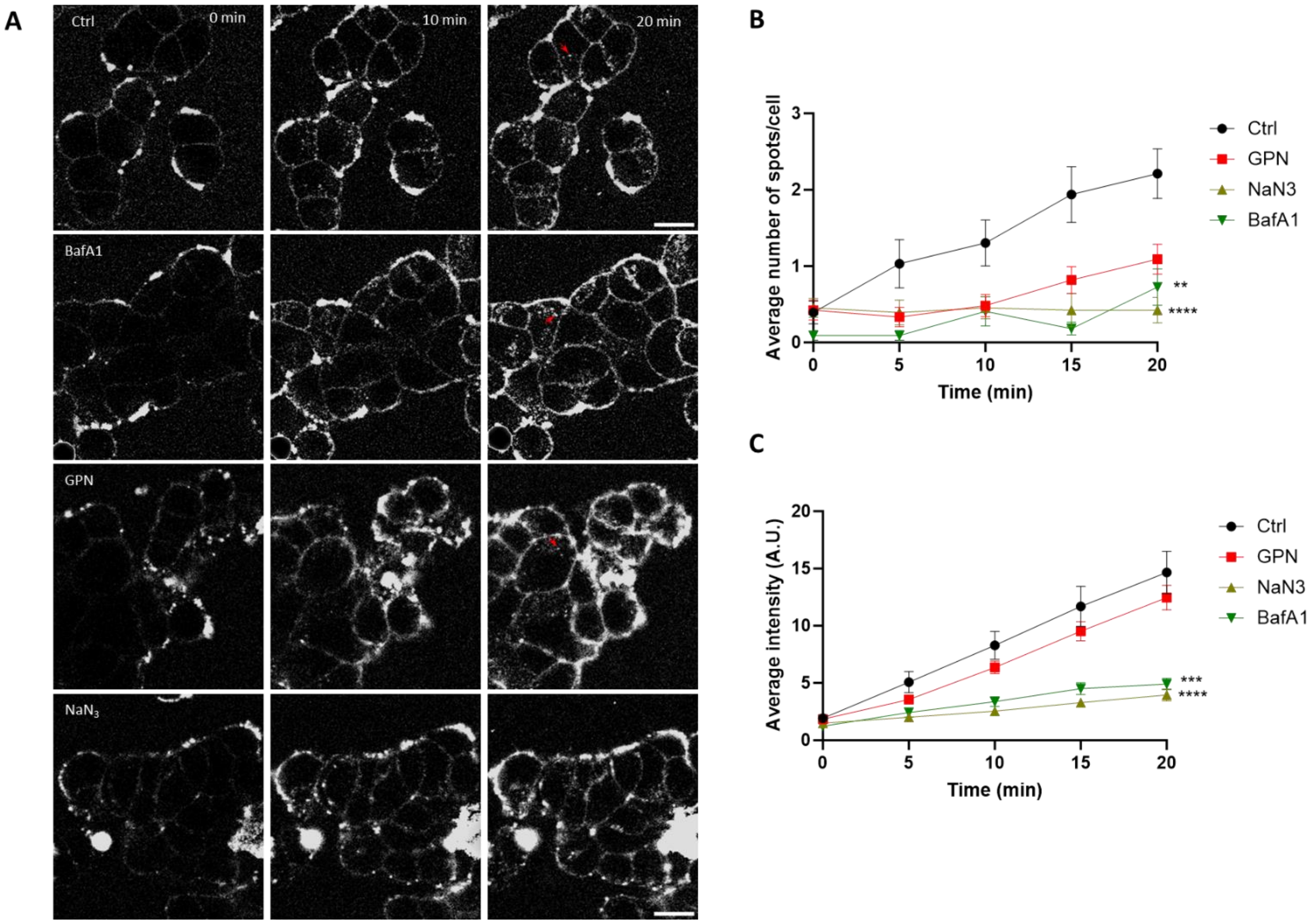

<u>Figure S3:</u> Penetration of AKH-αR5W4^{RhodB} in PC12 cells is ATP dependent. (A) Cells were treated were pretreated for 1h in Locke solution containing 150 µM sodium azide (NaN_3), 5 µM glycyl-l-phenylalanine 2-naphthylamide (GPN), 1 µM Bafilomycin A1 (BafA1), or with Locke solution at 4°C, before a pulse of 1 min with 5 µM AKH-αR5W4^{RhodB}. Images were acquired every minute at 1024 X 1024 pixel size. Scale bar: 10 µm. (B) Quantification of the number of vesicles and (C) Average cell intensity per cell containing AKH-αR5W4^{RhodB} over 20 min. A non-parametric Kruskal–Wallis with Dunn's multiple comparisons test was performed, $*p < 0.01$, $**p < 0.001$ and $****p < 0.00001$. The data corresponds to the mean ± SEM, of three independent experiments with > 30 cells analyzed.

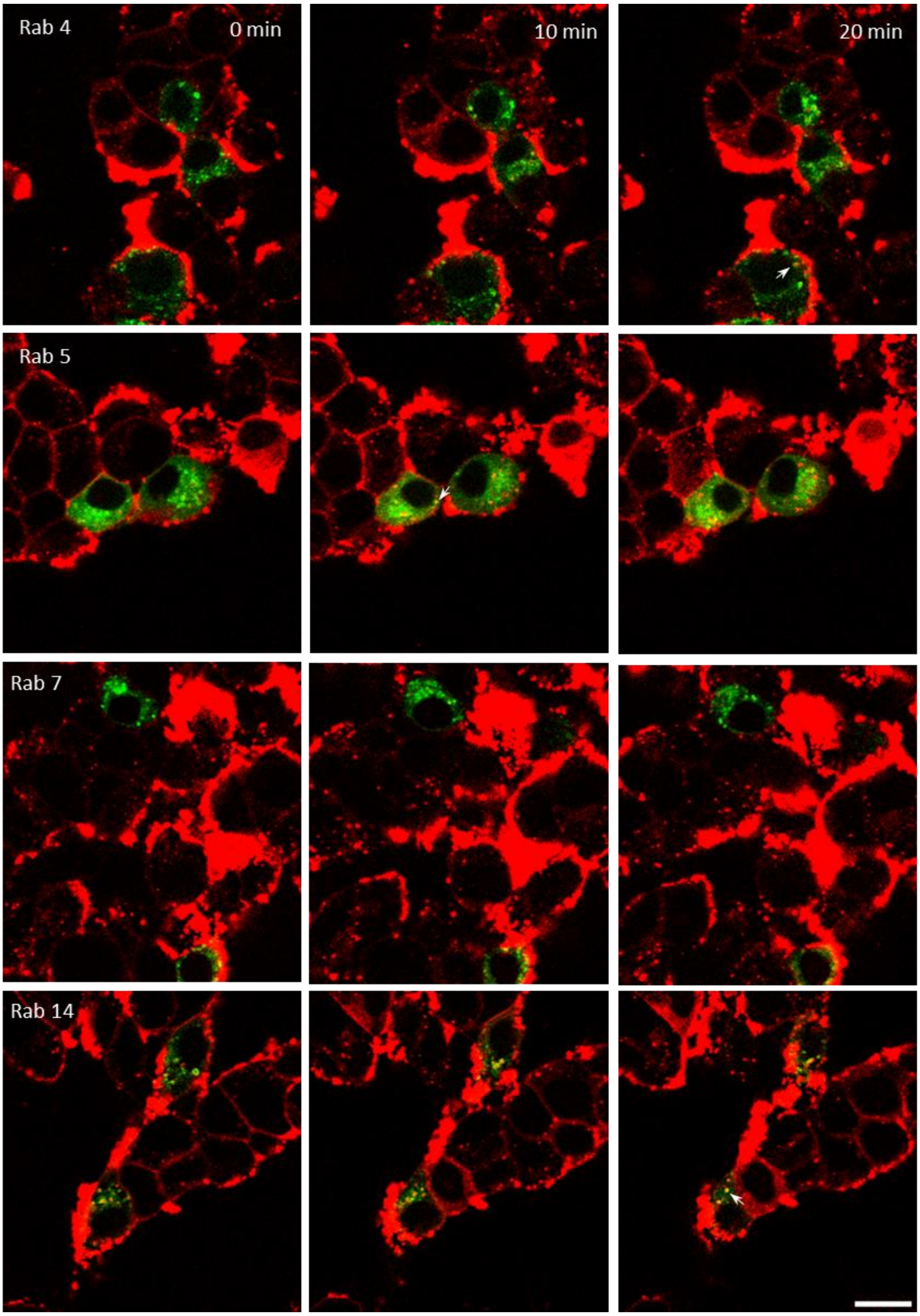

Figure S4: AKH-αR5W4^{RhodB} penetrates PC12 cells through the classical endocytosis pathway and is detected in the Golgi apparatus. Representative images of the colocalization between AKH-αR5W4^{RhodB} and exogenous Rab-GFP protein. Cells underwent a pulse for 5 min with 5 µM of AKH-αR5W4^{RhodB} , Images were acquired every minute at 1024 X 1024 pixel size. Arrows indicate vesicles with colocalized signal. Scale bar: 10 µm.
